# Correction: Shoombuatong, W., et al. iQSP: A Sequence-Based Tool for the Prediction and Analysis of Quorum Sensing Peptides via Chou’s 5-Steps Rule and Informative Physicochemical Properties. *Int. J. Mol. Sci.* 2020, *21*, 75

**DOI:** 10.3390/ijms21072629

**Published:** 2020-04-10

**Authors:** Phasit Charoenkwan, Nalini Schaduangrat, Chanin Nantasenamat, Theeraphon Piacham, Watshara Shoombuatong

**Affiliations:** 1 College of Arts, Media and Technology, Chiang Mai University, Chiang Mai 50200, Thailand; phasit.c@cmu.ac.th; 2 Center of Data Mining and Biomedical Informatics, Faculty of Medical Technology, Mahidol University, Bangkok 10700, Thailand; nalini.sch@mahidol.edu (N.S.); chanin.nan@mahidol.edu (C.N.); 3 Department of Clinical Microbiology and Applied Technology, Faculty of Medical Technology, Mahidol University, Bangkok 10700, Thailand; theeraphon.pia@mahidol.ac.th

The authors wish to make the following corrections to this paper:

Please note that all references have been re-numbered in the corrected paper.

The authors would like to apologize for any inconvenience caused to the readers by these changes.

1. Change in Title
  iQSP: A Sequence-Based Tool for the Prediction and Analysis of Quorum Sensing Peptides via Chou’s 5-Steps Rule and Informative Physicochemical Properties
  The correct title should be: iQSP: A Sequence-Based Tool for the Prediction and Analysis of Quorum Sensing Peptides using Informative Physicochemical Properties
2. Changes in Main Body Paragraphs

“a series of recent publications [67–72]…”

should be changed to

“a series of recent publications [67,70–72]…”

“As described in [35,85] and investigated by Equation (50) of [86], among those testing methods, the jackknife test is considered as one of the most rigorous that can provide a unique result for a given benchmark dataset.”

should be changed to

“Among those testing methods, the jackknife test is considered as one of the most rigorous that can provide a unique result for a given benchmark dataset [35].”

“into an unprecedented revolution [34,94–107].”

should be changed to

“into an unprecedented revolution [34].”

“S-palmitoylation sites in proteins [68], lysine crotonylation sites [108], and phosphotyrosine sites [109]. FurthermoreermPleore, our method could be integrated with other beneficial peptide features such as pseudo amino acid composition [110–112] or amphiphilic pseudo amino acid composition as proposed [113] by Chou [35,114] for further improving the QSP prediction.”

should be changed to

“S-palmitoylation sites in proteins, lysine crotonylation sites, and phosphotyrosine sites. Furthermore, our method could be integrated with other beneficial peptide features such as pseudo amino acid composition or amphiphilic pseudo amino acid composition for further improving the QSP prediction.”

